# Value of evidence from syndromic surveillance with cumulative evidence from multiple data streams with delayed reporting

**DOI:** 10.1038/s41598-017-01259-5

**Published:** 2017-04-26

**Authors:** R. Struchen, F. Vial, M. G. Andersson

**Affiliations:** 1 Veterinary Public Health Institute, Vetsuisse Faculty, University of Bern, Schwarzenburgstrasse 155, 3003 Bern, Switzerland; 2 Federal Food Safety and Veterinary Office, Schwarzenburgstrasse 155, 3003 Bern, Switzerland; 3 Department of Chemistry, Environment and Feed Hygiene, National Veterinary Institute (SVA), SE-751 89 Uppsala, Sweden; 4 Epi-Connect, Djupdalsvägen 7, SE-14251 Skogås, Sweden

## Abstract

Delayed reporting of health data may hamper the early detection of infectious diseases in surveillance systems. Furthermore, combining multiple data streams, e.g. aiming at improving a system’s sensitivity, can be challenging. In this study, we used a Bayesian framework where the result is presented as the value of evidence, i.e. the likelihood ratio for the evidence under outbreak versus baseline conditions. Based on a historical data set of routinely collected cattle mortality events, we evaluated outbreak detection performance (sensitivity, time to detection, in-control run length) under the Bayesian approach among three scenarios: presence of delayed data reporting, but not accounting for it; presence of delayed data reporting accounted for; and absence of delayed data reporting (i.e. an ideal system). Performance on larger and smaller outbreaks was compared with a classical approach, considering syndromes separately or combined. We found that the Bayesian approach performed better than the classical approach, especially for the smaller outbreaks. Furthermore, the Bayesian approach performed similarly well in the scenario where delayed reporting was accounted for to the scenario where it was absent. We argue that the value of evidence framework may be suitable for surveillance systems with multiple syndromes and delayed reporting of data.

## Introduction

Timeliness is a key measure of any public health surveillance system. System users and decision makers depend on it to take appropriate action based on the urgency and the type of responses required by the situation. As such, it should be assessed regularly^1^.

Most classical surveillance algorithms (including Salmon *et al*.^2^; Farrington *et al*.^3^; Noufaily *et al*.^4^) look for peaks with unusually high number of reported syndromic cases within a particular time period (e.g. a week), and generate an alarm if the counts exceed the threshold. Albeit simple, the approach has limitations that may hamper sensitivity and timeliness. Delays in the reporting of syndromic cases may result in counts remaining below a defined threshold until a majority of cases are reported, resulting in a delay or even failure of outbreak detection.

Delays in surveillance data may originate from an intrinsic biological process (e.g. incubation period) or from external processes (e.g. transport delay of the sample to the laboratory)^5^. While delays originating from the former cannot be reduced, analyses may be hindered by delays in case reporting decreasing the overall timeliness and usefulness of the early-warning surveillance systems^6^. Reporting delays depend, among other things, on statutory reporting regulations; on whether an electronic reporting system is in place; on the disease(s) under surveillance (whose identification process may be more or less complex); and on reporting units (e.g. different laboratories)^7^. Time lags between disease onset and notification can be estimated in terms of weeks (mostly as a result of lag between onset and diagnosis) even for notifiable disease reports (e.g. in Korea^8^, in the UK^9^). Reporting delays may be monitored to detect trends, for example following an intervention aimed at improving reporting timeliness^10^ or following a change in case definition^11^. They may also be modelled in order to better understand the factors leading to increasing time between disease onset and notification to the health systems^5, 12^.

The statistical interest in modelling delays in surveillance data is not new, but has so far mainly focused on the development of methods to obtain valid estimates of recent disease incidence^13^. Indeed, large reporting delays (e.g. as may be seen in cancer registries) may produce downwardly biased incidence trends, especially in the most recent years, when case ascertainment or reporting is subject to delays^14^. In the context of outbreak detection, delays occurring in a short time-window (on a scale of days or a few weeks) are more relevant than data with longer delays as they cannot be acted on promptly^9^. Accounting for reporting delays in outbreak detection algorithms (e.g. in syndromic surveillance SyS), is not trivial. This is, partly, the reason why most surveillance systems use the date of the reception of data, rather than the (often unknown) date of the health event itself. The main drawback of this common approach is the resulting reduction in sensitivity and specificity of the system^15^. In the relatively few systems for which all dates are known, a correction factor can be imputed based on mathematical models adjusting for the under-reporting bias owing to the time lag of the reporting process^16^.

Another difficulty may arise when faced with slowly increasing outbreaks. The number of cases reported in each time unit (e.g. each week or each day) may be too small to trigger an alarm. If the baseline is recalculated iteratively as in Noufaily *et al*.^4^, this may also result in outbreak- related cases being incorporated into the baseline. Guard bands leaving a short time lag between the current value under evaluation and the baseline have been used in order to reduce this risk of baseline contamination^17–19^.

Finally, many diseases cause more than one syndrome and combining data streams^20^ may result in increased sensitivity^21^. It is also desirable to combine the result from surveillance with other information. However, there is no straightforward approach when the algorithm is based on an alarm threshold^22^. Combining syndromic data from multiple data streams with other knowledge may be done within a Bayesian framework where the result is presented in the form of a posterior probability for a disease, or, when the hypotheses in the model are not exhaustive, as the odds for outbreak versus baseline. In Andersson *et al*.^23^ we proposed a framework where the result from SyS is expressed as the value of evidence in favour on outbreak, i.e. the likelihood ratio for the evidence under outbreak versus baseline conditions. This approach was evaluated using three syndromic indicators (nervous syndromes in horses, mortality in both horses and wild birds) for early detection of West Nile virus in France, achieving better performance in a multivariate than univariate system^24^. The most important difference between the value of evidence approach and classical SyS is that the former explicitly incorporates the assumptions about the disease of interest and also refers to these assumptions when the results are presented. By including in the model assumptions the distribution of syndromes under outbreaks and baseline conditions, it is possible to apply change point analysis to estimate the probability that the system is in outbreak or baseline conditions, and the most likely point of transition.

In this study, we show how the empirical Bayes^25^ likelihood ratio framework can be applied to perform change point analysis for multiple data streams and estimate the evidence accounting for delayed reporting of syndromes, using routinely collected cattle mortality data as an example.

## Methods

### The reporting system & reporting delay

Since 2000, it is compulsory for Swiss cattle farmers to report all births and deaths of animals on their holding to the “Tierverkehrsdatenbank” or TVD. Deaths on farm need to be reported within 3 days but the reporting of stillbirths is not compulsory (Animal Health Ordinance (AHO), SR 916.401). It is likely that some farmers report stillbirths as the birth of a calf that was alive and died after a few days^26^. For this reason, we termed perinatal mortality the sum of reported stillbirths and on-farm deaths within the first seven days after birth. We extracted all deaths on farm and perinatal deaths reported to the TVD between 01/01/2009 and 31/12/2011.

**Figure 1:**
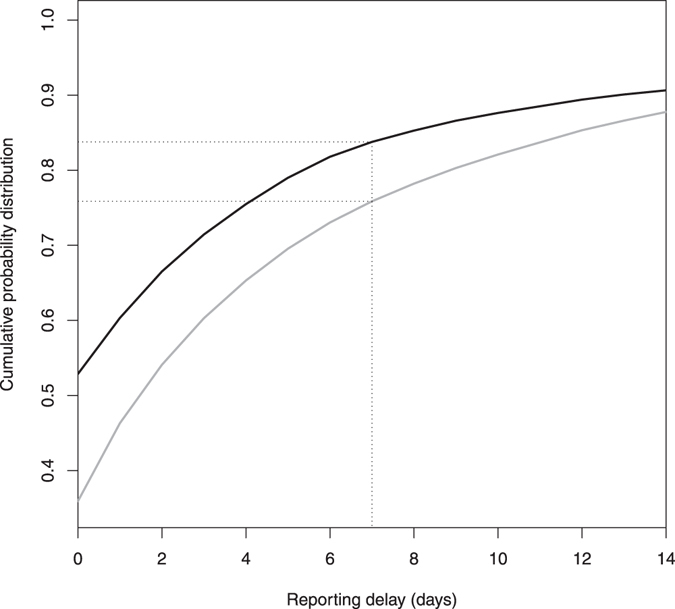
Cumulative probability distribution of the reporting delays for on-farm deaths (black) and perinatal deaths (grey) in the TVD. Over 80% of on-farm deaths and over 70% of perinatal deaths were reported within seven days of occurrence (dotted lines). We focused on reports with relatively short reporting delays (≤14 days) which are the most relevant for timely outbreak detection.

The time interval between the date the event occurred and the date it was reported to the TVD is termed reporting delay. It includes the time needed for the farmer to observe the event but may also include data entry errors. Reporting delay in the TVD has been previously assessed with a median of one day for deaths (range: 0–968) and two days for perinatal deaths (range: 0–907)^26^. Over 80% of deaths on farm and over 70% of perinatal deaths were reported within seven days of occurrence (Fig. 1). The focus of this study was on reports with relatively short reporting delays (≤14 days); since the minority of reports with longer delays (9 and 12% of on-farm death and perinatal death reports respectively) are less relevant for timely, “early” outbreak detection. Based on the cumulative probability distribution of the estimated reporting delays (Fig. 1), we used a binomial distribution to calculate for each day the number of cases occurring on day *t* that were reported on the same day (delay s = 0), 1 day later (s = 1), 2 days later (s = 2), etc. until all cases of day *t* were reported 14 days later (s = 14):

$${{\rm{R}}}_{{\rm{ts}}}\sim {\rm{Bin}}({{\rm{n}}}_{{\rm{ts}}},{{\rm{p}}}_{{\rm{ts}}})$$

where n_ts_ is the number of deaths occurring on day *t* if s = 0 or the number of deaths occurring on day *t* minus R_ts-1_ if s > 0, and p_ts_ is defined from the cumulative probability distribution of the reporting delays as the proportion of deaths c_ts_ occurring on day *t* that were reported on day s if s = 0 or as (c_ts_ − c_ts-1_)/(1 − c_ts-1_) if s > 0.

### The value of evidence from syndromic surveillance

We have previously proposed a tool for evaluating and presenting circumstantial ‘evidence’ of a disease outbreak from SyS, in which prior information and evidence (E) from the data are explicitly separated^23^. Applying Bayes’ theorem, the *a-posteriori* odds (O_post_) define our posterior belief about the disease state of the system given our prior belief and the syndromic evidence:

1 $${O}_{post}=\frac{P({H}_{1}|E)}{P({H}_{0}|E)}=\frac{P(E|{H}_{1})}{P(E|{H}_{0})}\ast \frac{P({H}_{1})}{P({H}_{0})}$$

Where:

- H_1_ is our hypothesis of interest (system is experiencing an outbreak of a specific disease of a group of diseases producing similar syndromes),
- H_0_ is the “null hypothesis” (the system is operating under baseline, non-outbreak, conditions),
- E is the evidence represented by a set of vectors with reported cases of (a) syndrome(s) (cattle deaths on farm and perinatal deaths in our case).

In reality, the probability of observing a given number of syndrome(s) is not constant throughout an outbreak and the appearance of different syndromes may not be simultaneous. If, for example, syndrome A usually appears before syndrome B, the absence of syndrome A will speak against an outbreak when a peak in syndrome B is observed. However, when a peak of syndrome A is observed, the absence of B does not speak against an outbreak at an early stage.

Our previous model evaluated evidence from only one day or week at a time^23^. In this study, we extend the framework to accumulate evidence over *n* points in time (30 days in this case).

**Figure 2:**
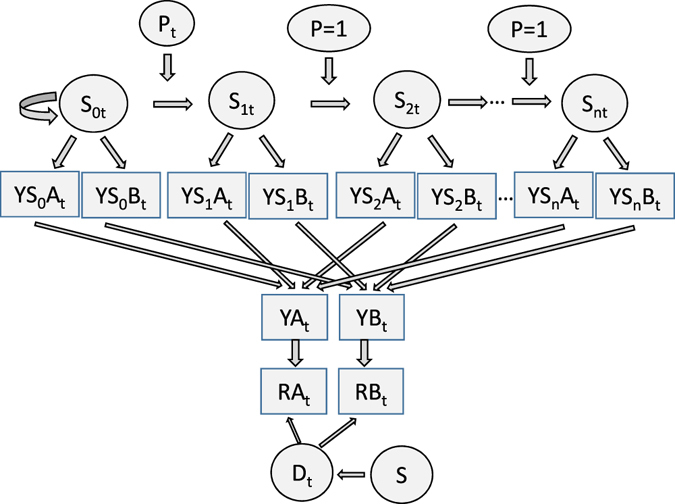
Representation of the system as an n + 1 state Hidden Markov Model. P_t_ is the probability of transition from state S_0_ to S_1_ at time t. S_it_ is the probability of the model being in state i at time t. YS_i_A_t_/B_t_ is the number of observed cases of syndrome A and B emitted by state i at time t. D_t_ is the probability that a syndrome observed at time t was reported at the time of observation (s). Finally, RA_t_/B_t_ is the number of observed syndromes that was reported from time t as seen on day s.

In order to estimate the evidence in favour of an outbreak, we consider H_1_ being composed of *n* sub-hypotheses H_11_… H_1n_, representing an outbreak being in its first 1 to *n* days. The system may be represented as an *n* + 1 state Hidden Markov Model (Fig. 2), where state S_0_ corresponds to hypothesis H_0_, no outbreak, and states S_1_ to S_n_ corresponds to the sub-hypotheses H_11_… H_1n_. The probability of an outbreak starting, that is a transition from state S_0_ to S_1_, is non-constant (i.e. seasonal). We set it so that outbreaks were more likely to occur in summer. Therefore, the prior probabilities P(S_i_) of the system being in each state S_i_ (i = 0:*n*) also vary.

The probability distributions for the number of reported syndrome cases under a non-outbreak situation and for an outbreak in state *n*, in combination with the prior probability for each state, were used to derive P(E|H_1_) and P(E|H_0_). H_0_ is the hypothesis that no outbreak is ongoing (S = 0) and H_1_ is the hypothesis that an outbreak is ongoing (S = 1:*n*).

The posterior probability of each hypothesis and the cumulative probability of an ongoing outbreak were obtained by numerically calculating the marginal probability of evidence, P(E), given the vector of prior probability of introduction. Marginal probability of evidence P(E) at a given time is defined as:

2 $$P(E)={\sum }_{i=0}^{30}P({S}_{i})P(E|{S}_{i})$$

where P(S_i_) is the prior probability of state S_i_ at time t. Posterior probability of state S_i_ (post(S_i_)) can subsequently be calculated using the formula

3 $$post({S}_{i})=P\frac{(S)P(E|{S}_{i})}{P(E)}$$

The value of evidence (V) in favour of an ongoing outbreak at each time *t* is defined as the Bayes’ factor, that is the ratio between the posterior and prior odds for H_1_ versus H_0_:

4 $$V=B=\frac{\sum _{i=1}^{30}P({S}_{i}|E)/P({S}_{i=0}|E)}{\sum _{i=1}^{30}P({S}_{i})/P({S}_{i=0})}$$

### Modelling the data

Seasonal variation in syndromes under baseline conditions was modelled by fitting negative binomial (NB) distributions to each of the two pre-processed^27^ mortality time series using dynamic binomial regression based on maximum likelihood (ML) in R with function *glm.nb* (package {MASS^28^}). The fitted models were used for data simulation and generating input parameters in the Bayesian model. First, a set of 200 baseline time series was generated from each of the two fitted NB models. Second, a set of 200 non-specific disease outbreaks of two different magnitudes (further referred to as smaller and larger outbreaks, see Supplementary Fig. S1) resulting in daily excess mortality cases of both syndromes was simulated and added to the simulated baseline counts. Details of the NB models and the baseline and outbreak simulation procedure are provided in the Supplementary Information.

In our previous work^23^, the distribution of baseline and outbreak-related syndrome cases was modelled directly using the NB distribution obtained from the regression (a similar method is also used in Salmon *et al*.^2^). The total outbreak distribution, i.e. the probability of observing *n* cases from the sum of baseline distribution and outbreak-related distribution, was calculated by numerical integration. However, the time to compute the distribution is proportional to the square of the maximum number of counts that result in very long computational time as the maximum number of counts increases. In this work, the number of counts at outbreak and baseline conditions is a magnitude higher calling for a faster, approximate solution.

To speed up calculations, the NB distributions were approximated with a (truncated) normal distribution with a standard deviation proportional to the mean.

P(base) ≈ TrunkNormal(exp_st_, var_st_)

Where

Exp.base_si_ is the expectation value from the NB model for syndrome s on day *i*

var_si_ = (exp.base_st_ ∙ sd.rel_s_)^2^.

sd.rel is the relative standard deviation for each syndrome calculated as:

5 $$sd{\rm{.}}re{l}_{s}=\sqrt{{\sum }_{t=1}^{t\,{\rm{\max }}}\frac{{({y}_{st}/{\exp }_{st}^{-1})}^{2}}{t\,{\rm{\max }}}}$$

The expected distribution of outbreak-related cases of syndrome s on day *i* of an outbreak

P(out) ≈ TrunkNormal(mean_si_, var_si_)

Where mean_si_ and var_si_ would ideally be based on expert judgement based on information on the disease and historical data. In the example, mean_si_ and var_si_ were calculated from sets of 1000 simulated outbreaks. For the ideal situation, the parameters were generated from a representative sample of simulated outbreaks, as they appear in the simulations. To investigate the effect of non-perfect expert knowledge, additional distributions were defined by calculating their mean and variance from biased samples of simulated outbreaks which differ in magnitude and progression rate (different parameters k, *µ* and *σ*, see Supplementary Information) from the outbreaks used in evaluation.

With this approximation, we computed the mean and variance of the total outbreak distribution (out.tot) for each possible combination of syndrome (s), time (t) and stage of the disease (i) analytically.

P(out.tot_sti_) = TrunkNormal(exp.tot_sti_, var.tot_sti_)

where

exp.tot_sti_ = exp.base_st_ + exp.out_si_

var.tot_sti_ = var.base_st_ + var.out_si_

However, the normal approximation results in erroneous probabilities at very low counts, since it implicitly assumes the possibility of negative number of outbreak-related syndromic cases. This will lead to artificially high values of evidence in favour of an outbreak on days when an extremely small number of counts are reported. This primarily happens when a reporting delay is present but not accounted for. To handle this, we introduced a heuristic 2-steps filtering algorithm:

- For each day, the algorithm finds the number of reported cases (nmin_ts_) that returns the minimum value of V (i.e. provides the strongest support against an ongoing outbreak);
- If the number of observed and reported cases (nobs_ts_) is smaller than nmin_ts_, it substitutes obs_ts_ with nmin_ts_.

This approximation effectively means that the cumulative probability for the left tail of the distribution of outbreak-related cases corresponding to negative counts is added to the probability of zero outbreak-related cases. The substitutions taking place were logged to estimate how frequent substitutions were and to confirm that it does not significantly impact the performance.

We compared the evolution of V for H_0_ and H_1_ in both syndromic time series before, during and after a set of simulated outbreak under three reporting scenarios:

- “Delay non-aware” scenario: the number of deaths occurring on day *t* is equal to the number of deaths reported on day *t* (i.e. regardless of when they truly occurred).
- “Delay aware” scenario: the number of deaths occurring on day *t* is estimated based on the number of deaths reported on day *t* and the probability distribution of the reporting delay.
- “No delay” scenario: all deaths are reported on the same day they occur and the number of deaths occurring on day *t* is equal to the number of deaths reported on day *t*.

### Evaluation of the framework

The performance of the system with regard to outbreak detection was compared among the three reporting scenarios using (1) the empirical Bayes approach introduced in this paper and (2) a classical process control approach adapted for reporting delay, as suggested by Salmon *et al*.^2^, and modified to handle multivariate data. For both methods, sensitivity and timeliness, measured as average time to detection, were calculated as a function of in-control average run-length (ARL), in practice by changing the threshold for the statistics (Log(V) or z-score) in small steps. In approach 1, the value of evidence, Log(V) of both syndromes for a particular day was compared to all potential thresholds between 0 (evidence neither in favour of or against outbreak) and 15 (evidence extremely strong in favour of outbreak), with ΔLog(V) = 0.125. An alarm was recorded if Log(V) exceeded these thresholds. In approach 2, the cumulative probability of the system producing the observed number of counts given the baseline condition was transformed to a z-score. To incorporate changes due to cases reported with delay, the z-scores of the last *d* days were considered, where *d* corresponds to the maximum reporting delay. For each day of observation, an alarm was recorded if the largest of these values exceeded a set of thresholds defined between 0 and 10, with Δz = 0.05.

Since the counts from the two syndromes in the example may be considered independent conditional on the season and outbreak status the combined z-scores for the two syndromes together was defined as:

6 $${z}_{tot}=\sqrt{{z}_{{A}^{\ast }}^{2}+{z}_{{B}^{\ast }}^{2}}$$

Where

z_A_ is z-score for perinatal deaths

z_A_ is z-score for on-farm deaths

z_A*_ = max(z_A_,0)

z_B*_ = max(z_B_,0)

The max() function ensures that a negative value of z_A_ or z_B_ will not result in a high z_tot_ thereby triggering an alarm.

Sensitivity was defined as the proportion of detected outbreaks among the total number of simulated outbreaks. An outbreak was considered as detected if an alarm was generated on at least one outbreak day. Time to detection was estimated for the outbreak period (i.e. outbreak start plus *n* days) and defined as the first day of outbreak detection (i.e. when an alarm was raised). In-control run length was estimated for the non-outbreak period (i.e. days *n* + 1 to 365) and defined as the first day with a false alarm. In case there was no alarm during the defined period, time to detection and in-control run length were set to 31 and 336 days, respectively.

## Results

**Figure 3:**
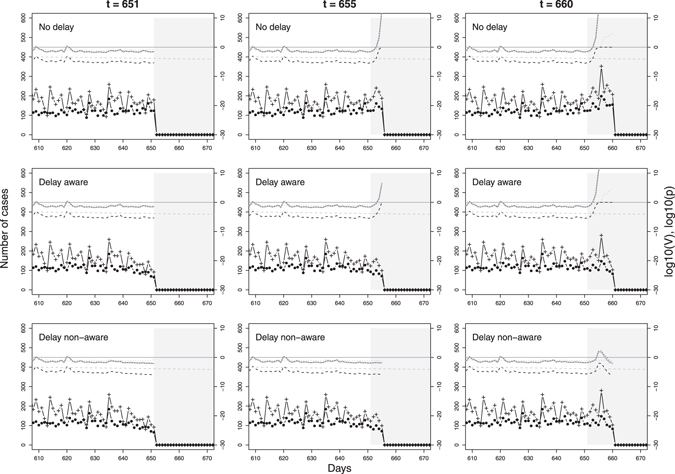
Evolution of the value of evidence at three days of observation (t) in comparison for the three scenarios no delay (top row), delay aware (middle row), and delay non-aware (bottom row). For illustration purposes, results for one specific outbreak, starting on day 651, were selected. Number of observed perinatal (black circles) and on-farm deaths (black pluses), value of evidence for both syndromes (solid grey line) and separately (grey and black dotted lines, respectively), prior probability that an outbreak is ongoing (grey dashed line) and posterior probability that an outbreak is ongoing given the evidence (black dashed line). The grey area represents the outbreak interval from days 651 to 678 while the horizontal grey solid line shows a value of evidence equal to 1, i.e. log10(V) = 0.

**Figure 4:**
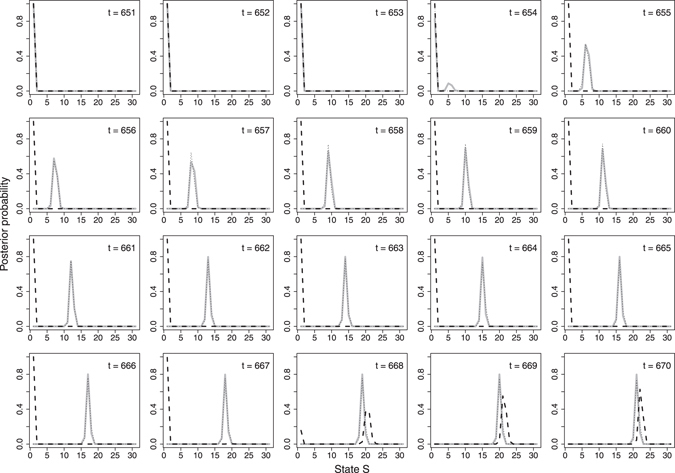
Posterior probability of being in state S (0–30) at a given day of observation (t) for the three scenarios no delay (grey solid line), delay aware (black dotted line) and delay non-aware (black dashed line). Twenty days of observation, starting on the first day of outbreak, were chosen to illustrate the transition of the system from baseline to outbreak condition.

The evolution of V based on the information available on the 1^st^, 5^th^ and 10^th^ day after the onset of a selected outbreak for each of the three reporting scenarios is presented in Fig. 3. For a larger outbreak starting on day 651. As a result of delayed reporting, daily counts of observed perinatal and on-farm deaths are lower for the most recent days in the two scenarios with reporting delay (middle and bottom row) compared to the scenario without reporting delay (top row). While V estimated for day 651 (left column) speaks against an outbreak for all scenarios, estimates for day 655 (middle column) show evidence in favour of an outbreak for both syndromes combined as well as for the on-farm deaths alone for the scenarios “Delay aware” and “No delay”. The development of V for the perinatal deaths alone highlights the importance of considering multiple syndromic data streams for outbreak detection, as it speaks in favour of an outbreak at a later stage (Fig. 3, right column, day 660) than on-farm deaths alone or both syndromes combined. The change in corresponding posterior probabilities of each state S_i_ for different days of observation is illustrated in Fig. 4 for all scenarios.

**Figure 5:**
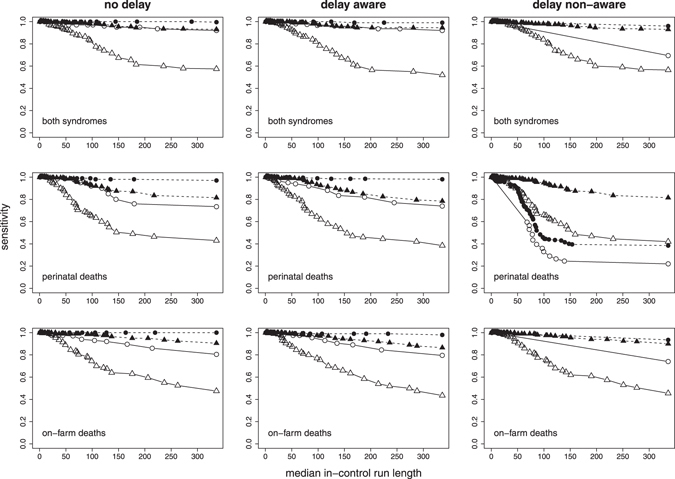
Sensitivity and median in-control run length for a range of alarm thresholds based on the value of evidence (circles) and the z-score (triangles), summarised for the smaller (empty symbols with solid lines) and larger (filled symbols with dashed lines) outbreak type. Values in the top right corner of the graph tend towards an ideal system with sensitivity = 1 (i.e. all outbreak signals are detected) and run length = 336 days (i.e. no false alarm occurs within 1 year).

**Figure 6:**
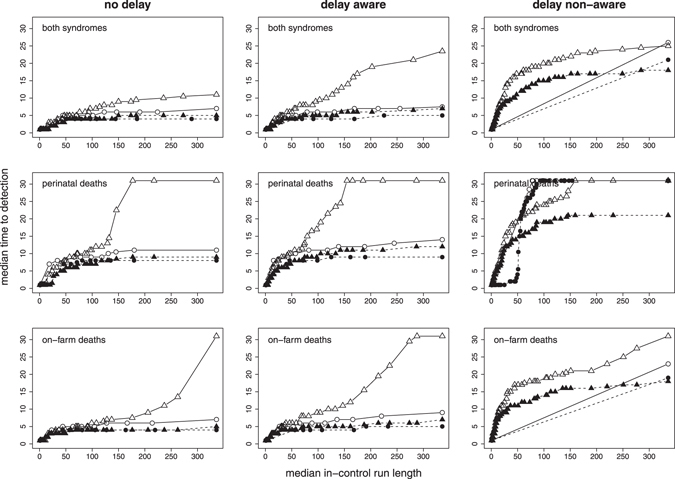
Median time to detection and in-control run length for a range of alarm thresholds based on the value of evidence (circles) and the z-score (triangles), summarised for the smaller (empty symbols with solid lines) and larger (filled symbols with dashed lines) outbreak type. Values in the bottom right corner of the graph tend towards an ideal system with a time to detection = 0 (i.e. an outbreak is detected on the day it starts) and run length = 336 days (i.e. no false alarm occurs within 1 year).

With a more restrictive alarm threshold (i.e. when only large values of V and z-scores raised an alarm), sensitivity and median time to detection generally impaired whereas in-control ARL improved (i.e. decreased). This trade-off between in-control run length and sensitivity or time to outbreak detection, respectively, is illustrated in Figs 5 and 6. Under the V-based approach, the performance of the system was comparable for the no delay and the delay aware scenarios. Under the z-based approach, however, the system’s performance in the delay aware scenario considerably decreased with regard to time to detection of the smaller outbreak type. The alarm thresholds resulting in a maximum in-control run length of 336 days (i.e. no false alarm occurred within 1 year) were less restrictive for the delay aware than the no delay scenario under the V-based approach, in contrast to the z-based approach with more restrictive thresholds (Table 1).

**Table 1:** Performance of the system under the V- and z-based approach, given the lowest alarm threshold resulting in a maximum in-control run length of 336 days.

	smaller outbreak			larger outbreak		
	threshold	sensitivity	time to detection	threshold	sensitivity	time to detection
V						
no delay	1.250	0.920	7	1	0.995	4
delay aware	1.125	0.920	7.5	0.875	0.990	5
z						
no delay	3.650	0.575	11	3.650	0.930	5
delay aware	3.900	0.520	23.5	3.900	0.945	7

**Figure 7:**
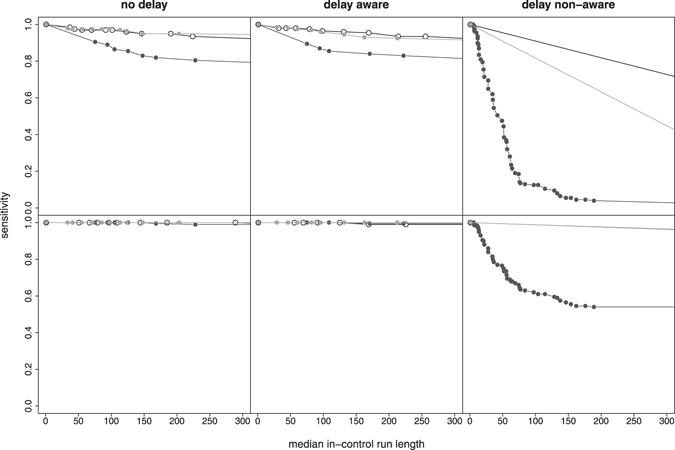
Influence of the choice of expert knowledge on the performance (sensitivity, in-control ARL) of detecting smaller (top row) and larger (bottom row) outbreak types under the V-based approach. Perfect expert knowledge was based on an ideal distribution of expected outbreak-related mortality cases (empty circles) for the given outbreak type. Non-perfect expert knowledge was simulated with expected outbreak distribution being biased either towards larger, steeply increasing outbreaks (black circles) or smaller, slowly increasing outbreaks (grey circles). Results are shown for combined mortality syndromes only.

**Figure 8:**
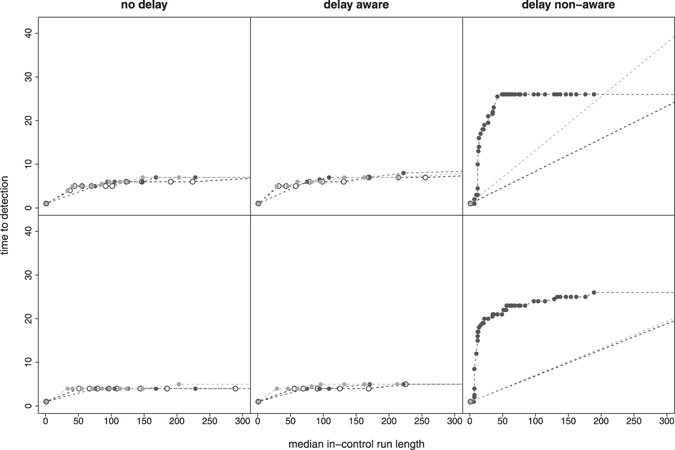
Influence of the choice of expert knowledge on the performance (median time to detection, in-control ARL) of detecting smaller (top row) and larger (bottom row) outbreak types under the V-based approach. Perfect expert knowledge was based on an ideal distribution of expected outbreak-related mortality cases (empty circles) for the given outbreak type. Non-perfect expert knowledge was simulated with expected outbreak distribution being biased either towards larger, steeply increasing outbreaks (black circles) or smaller, slowly increasing outbreaks (grey circles). Results are shown for combined mortality syndromes only.

Using non-perfect expert knowledge, i.e. biased distributions of expected outbreak-related mortality cases, mainly affected the ability to detect smaller outbreaks as shown in Fig. 7. Erroneously expecting large, steeply increasing outbreaks (black circles) decreased sensitivity of the smaller outbreak type in comparison to results based on perfect expert knowledge (white circles). In contrast, sensitivity of the larger outbreak type was comparable for all (ideal and biased) distributions. With regard to time to detection, outbreak detection performance was not substantially affected by the choice of expected outbreak distributions (Fig. 8).

## Discussion

Our results indicate that disease outbreaks can be detected much earlier and with only a marginal loss of specificity when incorporating delayed reporting compared to a system where reporting delay is present but not considered. Furthermore, the performance is on a comparable level to an ideal system in which no reporting delay is present, i.e. all cases are reported on the day they occur.

Moreover, the accumulation of evidence from several days resulted in a significantly better outbreak detection timeliness, at a given specificity; or a similar timeliness, but higher specificity, compared to an algorithm that, analogous to Salmon *et al*.^2^, only looks for days with unusual high number of counts. We expect that this pattern will be even more pronounced for outbreaks that initially produce a moderate number of outbreak-related counts. If, for example, we expect a rather flat (i.e. slowly propagating) outbreak, the threshold based on P(E|H_0_) may need to be set at a low level, resulting in many false alarms. On the other hand, a higher threshold could only be used for spike-like (i.e. rapidly propagating) outbreaks. The method based on the value of evidence might be more appropriate for timely detection of flat outbreaks since it is computed by considering the evidence of the past d days. In the example, we used a threshold for V as the alarm trigger, allowing for a comparison with the z-based approach. As discussed in Andersson *et al*.^23^, it is also possible to set a threshold for the posterior probability and use information about the expected seasonality of the disease of interest to further optimize the algorithm.

The effect of delayed reporting in SyS was assessed in a Bayesian framework by Salmon *et al*.^2^ using a similar approach to ours. However, while the authors used a full Bayes approach^20^ to model the baseline distribution and impact on delayed reporting, the SyS model is basically a Shewhart plot for which, in each time step, the count is compared with a threshold based on quantiles of the baseline distribution. In comparison, our work is an empirical Bayes approach^25^ where parameters for baseline and outbreak distributions and delayed reporting are estimated separately using classical methods. This information is then combined to estimate the posterior probability for an ongoing outbreak or the odds between outbreak and baseline. As proposed in Andersson *et al*.^23^, we separate the prior probability and the strength of the signal from SyS by estimating the value of evidence, i.e. the odds ratio between prior and posterior odds which is a proxy for the likelihood ratio (LR) for the observed evidence under the competing hypothesis (H_1_ ongoing outbreak and H_0_ baseline conditions).

Whereas neither approach can be claimed to be generally superior, they fulfil different niches. The approach of Salmon *et al*.^2^ is intended for automatic analysis of a large number of data streams with little human intervention, in which case it may be impractical to formulate assumptions for each possible disease. The disadvantage is an output that may not always be very informative. On the contrary, our approach is designed to provide comprehensive decision support when SyS is implemented for surveillance of a moderate number of specific diseases or classes thereof. By building in empirical knowledge from previous outbreaks in the form of probability distributions, we can present the user with a clearer picture of the possible significance of a peak. In veterinary SyS, the number of data streams is generally small, compared to human SyS. In this situation, it may no longer be optimal to focus on algorithms that can be easily automatized. Rather, we argue, that one should incorporate as much knowledge as possible in the system to make maximum use of the data that is available.

Several approaches for constructing control charts based on cumulative sum control chart (CUSUM) or exponentially weighted moving average (EWMA) for multivariate data have been proposed, as reviewed by Frisén^29^. We do not doubt that, for example, multivariate CUSUM could be used to construct an algorithm with similar performance to ours. However, as discussed by Frisén^29^, there is not a general best method applicable to all situations. Factors such as the relative importance of variables and the expected order of change points for two variables following an event would still have to be accounted for^29^. The Bayesian approach offers a straightforward and transparent way of incorporating such information and generates a model that not only triggers an alarm but may also explain why the alarm was triggered and how strong the evidence is in favour of an outbreak.

At first glance, incorporating a prior distribution for the expected number of outbreak related counts may be deemed more subjective than a method that solely relies on how unusual the peak is. However, even in such cases, the definition of the alarm threshold and estimation of the sensitivity is typically performed by simulating outbreaks. Thus, the Shewhart method also makes use of implicit assumption about the size and shape of the outbreak distribution. In practice, the decision whether the detection of an unusual peak should be considered sufficient to trigger an alarm depends also on what we expect to see in case of an outbreak. We argue that explicitly defining the outbreak distribution makes the reasoning of the system more transparent. As indicated in Figs 7 and 8, the approach works also when there is a discrepancy between the actual and expected outbreak distribution although, as expected, specifying that the expected outbreaks are large and steep, will reduce the sensitivity to detect small flat outbreaks.

Although in this work, we exemplify with baseline and outbreak distributions modelled by truncated normal distributions, it is not a limitation of the framework. In practice, each syndrome would be modelled by a distribution that is appropriate for its data structure. For example, low count data with and without clustering may be modelled by Poisson and NB distributions respectively as in ref. 23. Bayesian methods for fitting time series data have been described elsewhere^2, 30^ and may be combined with the framework presented here by integrating over the posterior distribution of the model parameters when calculating p(E|H_0_). However, using the full posterior distribution rather than point estimates as input would result in increased complexity and computational time which may be a hurdle for implementation in operative surveillance. We believe that the relatively simple approach presented here may be a competitive alternative to methods like multivariate CUSUM.

## Electronic supplementary material

S1
