# Supplementary material for: Value of evidence from syndromic surveillance with cumulative evidence from multiple data streams with delayed reporting
Source: Sci Rep. 2017 Apr 26;7:1191. doi: 10.1038/s41598-017-01259-5 (PMC5430846; doi:10.1038/s41598-017-01259-5)
Supplement: Supplementary file 1 — S1 [file 41598_2017_1259_MOESM1_ESM.pdf]

Supplementary Information

Value of evidence from syndromic surveillance with cumulative evidence from multiple data streams with delayed reporting

Struchen R, Vial F, Andersson M G

Simulation of cattle mortality time series, outbreak signals and expert knowledge of expected outbreak distributions

To describe the behaviour of the two cattle mortality time series under baseline conditions (i.e. in the absence of an outbreak), a negative binomial (NB) regression model was used for on-farm deaths and perinatal deaths, respectively:

$counts_{on\text{-}farm}$ ~ dow + month + holidays + post-holidays + t + AR_5

$counts_{perinatal}$ ~ dow + month + holidays + post-holidays + t + AR_4 + offset(log(births))

where dow = day of the week, t = continuous time variable and AR_n = autoregressive process of order n. Final models were selected based on a forward selection procedure, including different temporal covariates, as described in a previous study[1].

No major disease outbreaks in the Swiss cattle population were known for the years 2009 to 2011. To avoid contamination of the baseline time series by temporal aberrations (which might represent unnoticed outbreak signals, but also extreme events such as e.g. heat waves, or excessive random noise), it was cleaned using an iterative method of model fitting and aberration removal[2,3]: temporal aberrations were identified in the observed data as those values exceeding the 95th percentile of the model that best fitted the perinatal time series. Such outlier values were then replaced by the value of this percentile. The procedure was repeated until outliers were no longer identified.

Estimated coefficients and standard errors of the final NB models are presented in Supplementary Table S1 while fitted baselines and 95% confidence intervals of the daily counts are shown in Supplementary Fig. S1.

Supplementary Table S1. Estimated coefficients and standard errors (SE) of the final negative binomial models.

	On-farm deaths		Perinatal deaths	
	Coefficient	SE	Coefficient	SE
Baseline	5.509007	0.064224	-2.873609	0.054441
Day of the week				
Tue	-0.291368	0.026190	-0.141990	0.014758
Wed	-0.334026	0.025363	-0.126643	0.014103
Thu	-0.385813	0.022631	-0.145792	0.013462
Fri	-0.296712	0.021984	-0.143661	0.013043
Sat	-0.541516	0.019771	-0.188263	0.012305
Sun	-0.885132	0.015952	-0.274578	0.012406
Month				
Feb	-0.032357	0.016534	-0.092790	0.015685
Mar	-0.100340	0.017828	-0.174794	0.019119
Apr	-0.210267	0.023857	-0.153343	0.026961
May	-0.312362	0.028147	-0.085142	0.029349
Jun	-0.354202	0.030457	-0.098250	0.030931
Jul	-0.306646	0.028496	-0.103286	0.025380
Aug	-0.251616	0.026301	-0.129420	0.020609
Sep	-0.214631	0.024362	-0.165931	0.015594
Oct	-0.170908	0.022034	-0.177597	0.014828
Nov	-0.118135	0.020032	-0.153208	0.015004
Dec	0.008138	0.016619	-0.018351	0.015004
Bank holidays	-0.481785	0.024498	-0.182266	0.022836
Post Bank holidays	0.233356	0.027638	0.108818	0.025101
t	-0.000021	0.000011	0.000042	0.000011
AR_1	0.000276	0.000145	0.000636	0.000255
AR_2	0.000228	0.000131	0.000296	0.000252
AR_3	0.000351	0.000128	0.000074	0.000254
AR_4	0.000044	0.000128	0.000640	0.000253
AR_5	0.000413	0.000130	-	-

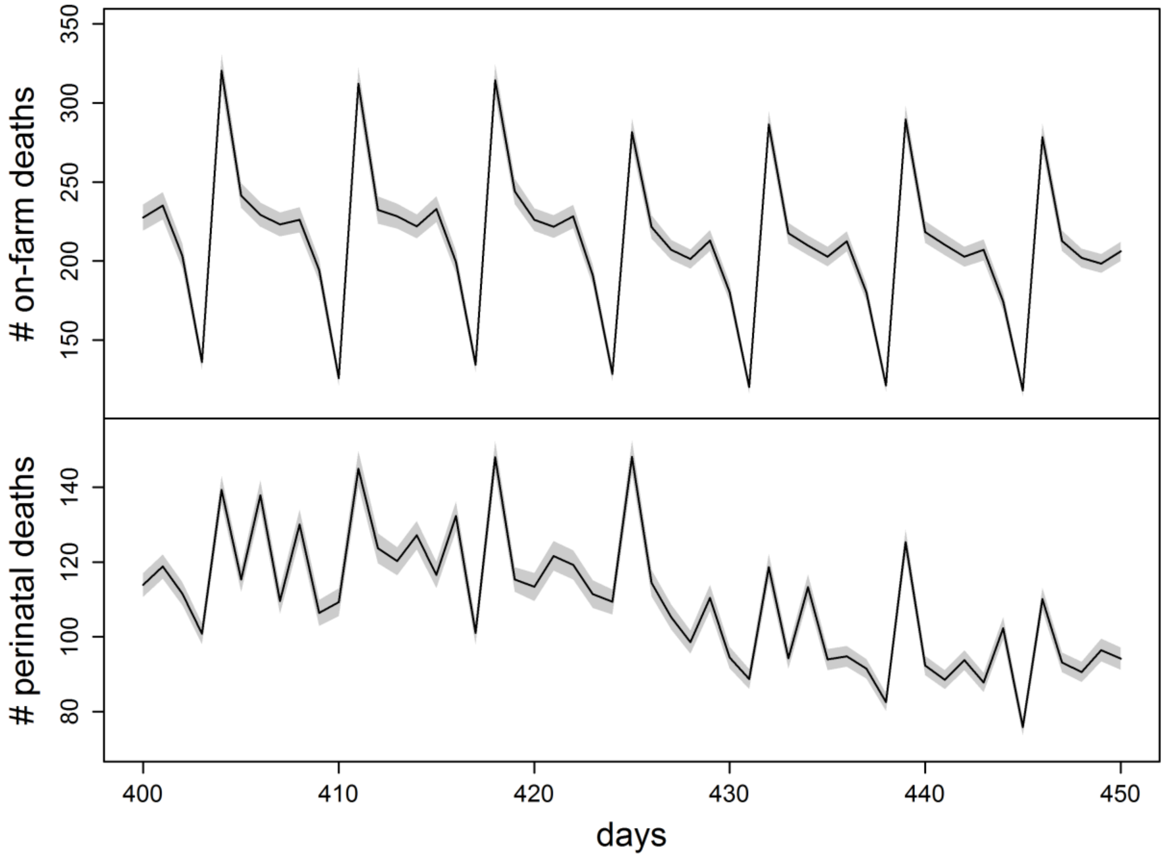

Supplementary Figure S1. Fitted baselines (black line) and 95% confidence intervals (grey area) of the daily number of on-farm and perinatal deaths. For visibility reasons, results are shown only for a small subset of the time series.

For both mortality syndromes, the resulting "outbreak-free" historical baselines were used to simulate a set of 200 baseline time series, each consisting of 1,095 days (three years). For each of these time series, the daily number of mortality events was randomly sampled from a NB distribution with a mean defined by the predicted value for the corresponding day of the model that was previously re-fitted to the cleaned historical baseline.

Non-specific disease outbreaks were simulated in two steps. First, the number of infected animals was generated in a similar way as presented by Noufaily et al.[4] and adopted by Vial et al.[5] for meat inspection data. Each baseline time series was equally divided into 1) a pre-outbreak period; 2) an outbreak period; and 3) a post-outbreak period. The start of the

outbreak at day t was randomly sampled among the outbreak period (from days 366 to 730). The probability of outbreak occurrence varied seasonally (Supplementary Fig. S2), being largest during summer which may be the case for e.g. vector-borne diseases. While the pre-outbreak period was used to evaluate the performance of the algorithm under baseline conditions, the post-outbreak period ensured that outbreaks starting at the end of the outbreak period could still be fully evaluated by the algorithm. The number of infected animals was randomly generated from a Poisson distribution with mean equal to a constant k times the standard deviation of the predicted baseline count at day t. The resulting total number of outbreak cases was then randomly distributed in time according to a lognormal distribution with mean μ and standard deviation σ. With increasing μ, the same number of outbreak cases for a given k was distributed over a wider time window. Based on a visual inspection of the resulting magnitude and duration of outbreaks when using different values for k (which mainly influences the total outbreak size) as well as the parameters μ and σ (which influence the temporal progression of the outbreak), two outbreak types of different magnitude $k = \{16, 31\}$, with $\mu = 2$ and $\sigma = 0.5$, were selected (further referred to as smaller and larger outbreaks respectively). For both types, a set of 200 outbreaks was simulated. Start, end, duration and size were recorded for each outbreak.

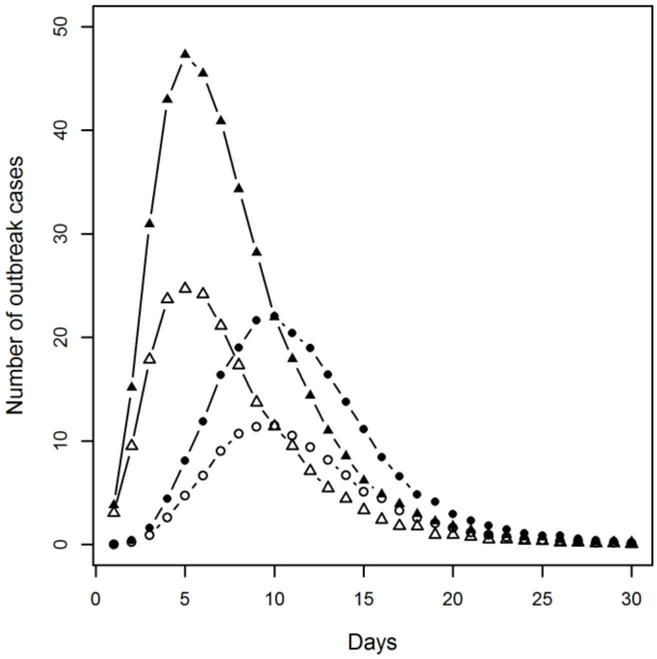

Supplementary Figure S2. Mean number of simulated daily outbreak cases of perinatal (circles) and on-farm (triangles) deaths, summarised for the smaller (empty symbols) and larger (filled symbols) outbreak type.

The proportion of infected animals that developed one of the two mortality syndromes was defined from the number of infected animals per day, with a proportion of 0.7 and 0.4 for on-farm and perinatal deaths, respectively. The daily outbreak cases of on-farm and perinatal deaths were randomly generated from a Poisson distribution with mean equal to the proportion of infected animals that developed the corresponding syndrome and inserted into a new copy of the 200 simulated baseline time series of each mortality syndrome (i.e. one outbreak per outbreak period per time series). Additionally, perinatal mortality cases were defined to occur with a delay randomly chosen between 1 and 7 days compared to on-farm deaths. Resulting outbreak types were illustrated in Supplementary Fig. S3.

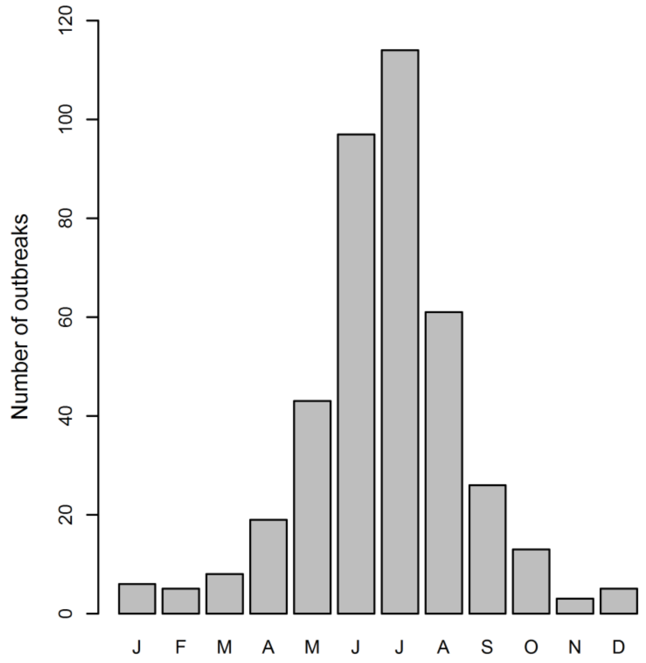

Supplementary Figure S3. Number of simulated outbreaks starting each month, illustrating the seasonality of outbreak probability during the outbreak period.

Parameters (mean and variance) to define expected distributions of outbreak-related cases (Supplementary Fig. S4) were derived from representative or biased sets of 1000 simulated outbreaks. To simulate ideal expert knowledge for the smaller and larger outbreak, respectively, mean and variance were calculated from a representative set of outbreaks simulated based on $\mu = 2$, $\sigma = 0.5$, and $k = 16$ or $k = 31$. To simulate non-perfect expert knowledge, mean and variance were computed from two biased sets of simulated outbreaks based on 1) $\mu = \{1;2\}$, $\sigma = 0.5$, and $k = \{10:40\}$; 2) $\mu = \{2.5;3\}$, $\sigma = 0.5$ and $k = \{10:40\}$.

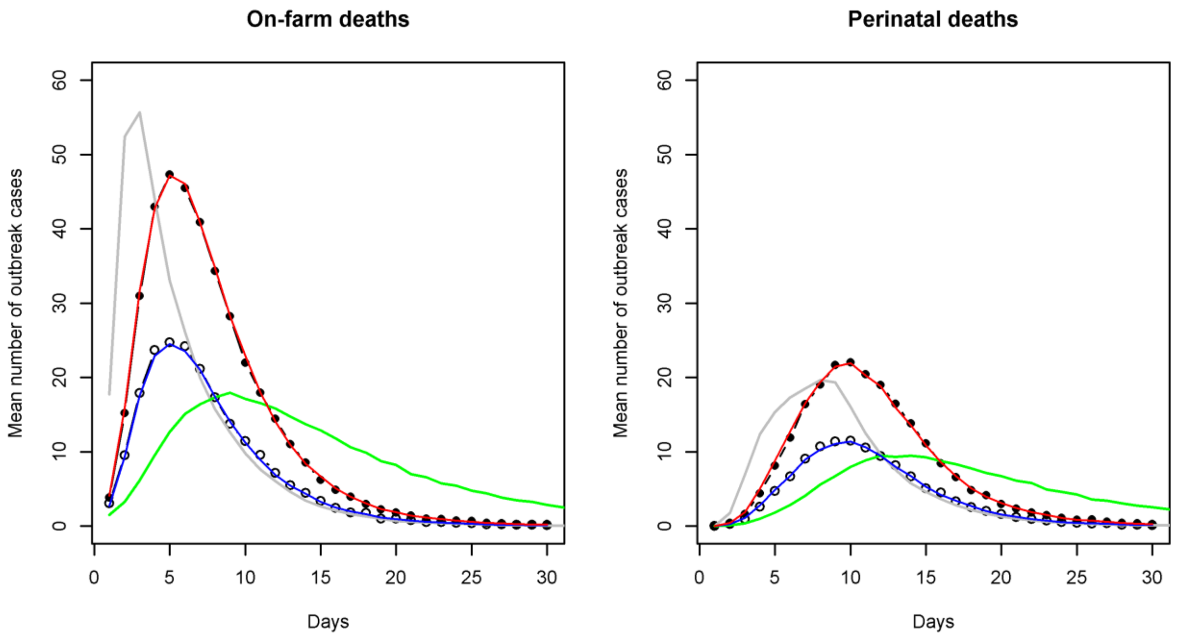

Supplementary Figure S4. Expected distributions of outbreak-related cases of on-farm (left) and perinatal (right) deaths, representing ideal (red and blue) and non-perfect (grey and green) expert knowledge. Mean shape of smaller (empty circles) and larger (filled circles) outbreaks are shown.

References

1. Struchen, R., Reist, M., Zinsstag, J. & Vial, F. Investigating the potential of reported cattle mortality data in Switzerland for syndromic surveillance. *Prev. Vet. Med.* **121,** 1–7 (2015).
2. Dórea, F. C. *et al.* Retrospective time series analysis of veterinary laboratory data: Preparing a historical baseline for cluster detection in syndromic surveillance. *Prev. Vet. Med.* **109,** 219–227 (2012).
3. Dupuy, C. *et al.* Pilot simulation study using meat inspection data for syndromic surveillance: use of whole carcass condemnation of adult cattle to assess the performance of several algorithms for outbreak detection. *Epidemiol. Infect.* 1–11 (2015). doi:10.1017/S0950268814003495
4. Noufaily, A. *et al.* An improved algorithm for outbreak detection in multiple surveillance systems. *Stat. Med.* **32,** 1206–22 (2013).
5. Vial, F., Thommen, S. & Held, L. A simulation study on the statistical monitoring of condemnation rates from slaughterhouses for syndromic surveillance: an evaluation based on Swiss data. *Epidemiol. Infect.* 1–11 (2015). doi:10.1017/S0950268815000989
